# Optimizing an App-Based Just-in-Time Adaptive Intervention for Stimulant Use Among Sexual Minority Men Living with HIV: Protocol for a Community-Engaged Research Approach and Hybrid-Experimental Design

**DOI:** 10.2196/76741

**Published:** 2025-12-02

**Authors:** K Marie Sizemore, Judith T Moskowitz, H Jonathon Rendina, Shannon Gray, Hannah Hyejin Park, Adam Carrico, Jonathan Avery, Brett Millar

**Affiliations:** 1 Department of Psychiatry Robert Wood Johnson Medical School New Brunswick, NJ United States; 2 Institute for Health, Health Care Policy and Aging Research Rutgers, The State University of New Jersey New Brunswick, NJ United States; 3 Department of Medical Social Sciences Northwestern University Chicago, IL United States; 4 Milken Institute of Public Health George Washington University Washington, DC United States; 5 Cooper University Health Care Cherry Hill, NJ United States; 6 Robert Stempel College of Public Health and Social Work Florida International University Miami, FL United States; 7 Addiction Psychiatry Weill Cornell Medicine New York, NY United States

**Keywords:** mHealth, EMA, just-in-time adaptive intervention, ecological momentary intervention, ecological momentary assessment, HIV, substance use disorder, stress, behavior change, mHealth

## Abstract

**Background:**

In the United States, sexual minority men (SMM) are disproportionately affected by HIV. For this population, intersecting sexual minority– and HIV-related stressors add to general life stress, increasing health risks. Stress is associated with HIV progression and is linked to transmission risk behaviors, such as medication nonadherence and substance use. Substance use is a particularly important risk factor for HIV transmission; beyond injection drug use, recreational use is associated with sexual transmission risk behaviors and an increased risk of HIV among SMM. Interventions targeting stress responses may be especially useful for HIV risk reduction among substance-using SMM (SUSMM) living with HIV (LWH). Positive affect interventions have shown promise in reducing stress in the context of chronic illness, including HIV. However, few studies have examined these interventions and their potential health benefits for SUSMM-LWH.

**Objective:**

This protocol describes a project (R34DA053999) that builds on our pilot work exploring the induction of positive affect as a potential intervention to reduce stress and improve health outcomes among SMM-LWH. This protocol aims to employ a community-engaged research approach, using the multiphase optimization strategy, to iteratively tailor our app-based positive affect intervention, TeaTime, for SUSMM-LWH.

**Methods:**

In phase 1, we enrolled 10 SUSMM-LWH into an open-phase pilot using our existing app-based ecological momentary intervention (EMI), which employs a just-in-time adaptive intervention design. We collaborated with a community advisory board to tailor our intervention content and app design using focus group feedback. In phase 2, we conducted a pilot factorial optimization trial to assess the acceptability and feasibility of adding 2 new features that may enhance intervention design and delivery. Specifically, we piloted a 2×2 factorial design, randomizing 80 SUSMM-LWH to receive either (1) random craving prompts or (2) smartwatch integration or both or neither. Analyses will examine group differences on feasibility and acceptability outcomes, as measured by the System Usability Scale and Mobile App Rating Scale, as well as retention and engagement.

**Results:**

The project was funded in February 2022, and data collection for phase 1 was completed in December 2023. Phase 2 was launched in July 2024, with data collection completed by August 2025. Results from phase 1 are expected to be submitted for publication by December 2025.

**Conclusions:**

In this protocol, we combine methods from social science, intervention science, and software development into a single innovative approach to enhance and optimize an app-based, just-in-time adaptive, EMI for SUSMM-LWH. Findings from this study will support the development of a fully optimized intervention ready for evaluation and implementation. In a future randomized controlled trial, the ecological momentary assessment design will also enable the collection of day-level data on intervention efficacy.

**International Registered Report Identifier (IRRID):**

DERR1-10.2196/76741

## Introduction

### Background

In the United States, gay, bisexual, and other sexual minority men (SMM) are disproportionately affected by HIV compared with the general population [1]. For SMM living with HIV (LWH), intersectional sexual minority and HIV-related stress add to general life stressors, increasing health risks [2,3]. Intersectional minority stress also includes experiences of discrimination among racial and ethnic minorities [4,5], who represent a majority of the SMM-LWH population [6,7]. Because of the cumulative impact of these intersecting stressors, SMM-LWH experience significant health disparities compared with the general population [8-10]. The “cumulative stress” hypothesis asserts that chronic and acute stressors in later life have an additive impact on vulnerabilities already programmed by early life adversity [11]. Cumulative stress is hypothesized to result in increased allostatic load, operationalized as a cumulative biological burden leading to poorer health outcomes (eg, HIV progression) [12]. Furthermore, research supports a bidirectional association between psychosocial stress and HIV progression [13].

Stress is not only linked to distal outcomes (eg, HIV progression) [13,14], but is also associated with proximal behavioral outcomes that increase the risk of transmission (eg, medication nonadherence and substance use) [15,16]. Substance use is a predictor of poor HIV medication adherence [17,18], and accordingly, substance-using (SU) individuals LWH are at higher risk for HIV disease progression [19,20]. Substance use is also a particularly important risk factor for HIV transmission, as recreational use is associated with other transmission risk behaviors (TRBs), and injection drug use carries a substantially higher risk of transmission compared with other TRBs. In addition to the risk associated with injection drug use among SMM, studies show that recreational substance use is associated with sexual TRB and an increased likelihood of HIV among SMM [21-23]. As such, interventions targeting stress responses may be particularly useful for HIV risk reduction among SUSMM-LWH.

Stress is also associated with increased substance use cravings [24,25]. Even minor stressful events have been linked to heightened substance use cravings [26]. Ecological momentary assessment (EMA) studies show that daily cravings are strong predictors of subsequent substance use [27,28]. Furthermore, research suggests that cravings may mediate the association between stress and substance use [29]. This may help elucidate findings from prior studies showing a stronger link between cravings and substance use than between stress and substance use [30]. It is also important to note that, in addition to cravings induced by stress, cravings can be cue-induced [31-33]. However, studies comparing stress-related and drug-related cues on cravings have found greater subjective emotional responses and cardiovascular activity under stress-related conditions [33].

Research shows that positive affect buffers against negative stress-related health outcomes [34-36]. Positive affect refers to experiences of positive moods such as joy, interest, and alertness [37], whereas mindfulness is a state of focused, nonjudgmental awareness of one’s thoughts, motivations, and behaviors [38]. Positive affect is associated with better outcomes in substance use [39] and HIV-related health [40]. Among individuals LWH, research indicates that positive affect is associated with increased odds of linkage to HIV care and treatment adherence [36]. One study also found that positive affect was linked to a significant decrease in mortality risk related to AIDS [41]. More generally, among people living with a chronic illness, positive affect interventions have shown significant effects in increasing medication adherence [42] and lowering reported stress levels related to diagnosis [43-45]. Positive affect has also been associated with lower levels of substance use [17]. Furthermore, mindfulness is positively correlated with positive affect among SMM-LWH [46].

### Description of the Initial Intervention

Moskowitz and colleagues [40,47] developed an integrative positive affect intervention for people experiencing health-related stress. The intervention is based on the revised stress and coping theory [45] and the broaden-and-build theory of positive emotion [48]. These theories describe how positive emotion supports coping and well-being by building social, intellectual, and physical resources. The intervention’s content, methods, protocol, and design have been published elsewhere [49]. Findings from a randomized controlled trial (RCT) demonstrate the effectiveness of this integrative intervention for people newly diagnosed with HIV [40]. The results show decreases in intrusive and avoidant thoughts related to HIV, along with increases in positive affect on a day-to-day basis [40]. Findings also indicate that the intervention’s effect sizes increase over time [40].

This intervention was recently tested with SUSMM-LWH [39,50]. Specifically, the study examined the efficacy of Moskowitz’s intervention in enhancing contingency management for SMM-LWH who use methamphetamine. The pilot RCT demonstrated the feasibility of administering the intervention with SUSMM-LWH and showed significant increases in positive affect [50]. In the full RCT, participants receiving the intervention exhibited significantly higher levels of mindfulness 3 months postintervention, along with decreases in cravings and self-reported drug use [39]. This work demonstrates the promise of implementing this integrative intervention with SUSMM-LWH and its potential effectiveness in improving substance use outcomes for this population.

While the in-person delivery of this program has been effective in improving health outcomes, there has been increased interest in remote intervention delivery. Moskowitz’s intervention has already been tailored for eHealth, web-based delivery [4,51]. In a recent study, the acceptability and feasibility of this web-based version were tested among individuals living with comorbid HIV and depression, and it was found to be both feasible and acceptable for this population [52].

### Just-In-Time Adaptive Intervention Ecological Momentary Intervention Development

More recently, our team sought to adapt this intervention for app-based delivery. Evidence demonstrates the feasibility of mobile interventions specifically among SMM-LWH [53]. Such interventions have shown suitability, acceptability, and efficacy among SMM-LWH, as they allow a component of privacy [53]. Mobile apps can also be used to deliver ecological momentary interventions (EMIs), which provide an opportunity to offer in-the-moment support to SUSMM-LWH [54]. EMIs, much like EMAs, are delivered to participants in their daily lives; however, instead of simply collecting data, EMIs also include an intervention component. Further, this intervention content can be triggered based on participant responses to the EMA, and in this way, the delivery of the intervention is tailored to when participants need it the most [54]. This customization of intervention delivery is often referred to as a just-in-time adaptive intervention (JITAI) design.

**Figure 1:**
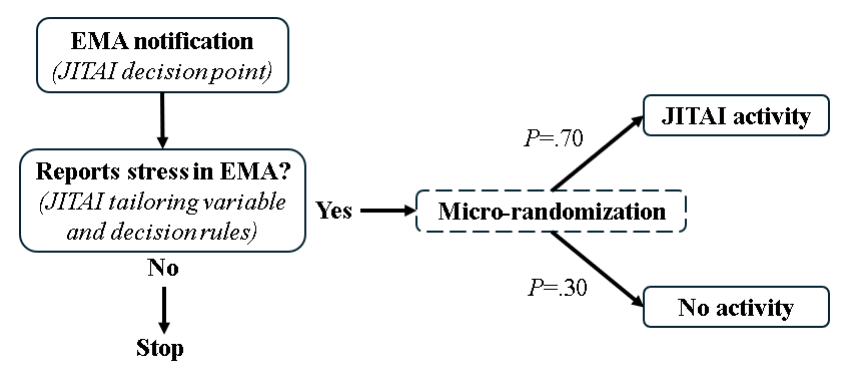
Just-in-time adaptive intervention (JITAI) design for micro-randomization in the proof-of-concept pilot. EMA: ecological momentary assessment.

In our proof-of-concept pilot, we adapted the evidence-based positive affect intervention’s web content [40] for mobile app delivery, using an EMI delivery format and JITAI design [55]. Our JITAI EMI included 7 modules designed for positive affect induction (eg, noticing and savoring positive events, acts of kindness, behavioral activation, personal strengths, positive reappraisal, and gratitude). In this pilot, we recruited 22 SMM-LWH to engage with the app over a 90-day period. Within the app, participants had access to “on-demand” (ie, user-initiated) intervention content, allowing them to review the skills at any time. The app also delivered a daily positive message accompanied by an image from a bank of motivational quotes, each referring to one of the intervention skills. Additionally, over the course of the 90-day study, participants received a daily EMA survey that included questions about stress, positive and negative affect, and medication adherence. Depending on their survey responses, participants were randomized (at a 2:1 ratio) to receive either a brief intervention activity or a control message. Specifically, if a participant reported feeling stressed in the EMA, they were randomized to receive 1 of 32 JITAI activities. This procedure was part of the micro-randomized trial (MRT) design, which we also tested in this proof-of-concept pilot study. Selection of the specific JITAI activity to be delivered occurred through a process of further micro-randomization. Once participants were randomized to receive a JITAI activity, they were further randomized, with equal probability (*P*=.14), to receive an activity from 1 of the 7 modules within the app. After being randomized to a specific module, the intervention activity to be delivered at that moment was randomly selected from a bank of activities within that module. See Figure 1 for a diagram depicting our JITAI design and micro-randomization process in this study. Findings from our proof-of-concept pilot suggest that our app-based JITAI EMI is an acceptable and feasible delivery mode for SMM-LWH [55].

### Objectives

However, we have not yet piloted our app with SUSMM-LWH. Holding an additional marginalized identity (ie, SU) presents unique stressors and additional barriers to intervention [56,57]. For example, individuals with substance use disorders often face stigma and discrimination from health care providers [58]. This added minority stress can have a multiplicative effect when compounded by other marginalized identities (eg, racial and sexual minority status, HIV status). The JITAI EMI design is especially promising for SUSMM-LWH, given their experience of multiple intersecting stressors throughout the day (eg, microaggressions, stigma, discrimination). Further, SUSMM-LWH may benefit from the on-demand and in-the-moment support offered by an app-based JITAI EMI. As such, we aim to tailor our existing intervention content and optimize our JITAI EMI design and delivery to reduce stress and substance use among SUSMM-LWH. We also aim to further enhance our intervention by examining the acceptability and feasibility of adding new features that may improve engagement, as well as refining the app’s overall design and delivery.

## Methods

### Overview of the Study Design

In phase 1, we conducted an open-phase pilot and engaged community members to help tailor our existing app for SUSMM-LWH. In phase 2, we piloted a randomized factorial optimization trial to assess the acceptability and feasibility of adding additional features (ie, smartwatch integration and random craving prompts) to optimize our JITAI EMI’s design and delivery. We note that this project was not funded as a clinical trial and was not powered to test intervention outcomes; rather, it was designed as an acceptability and feasibility study. Accordingly, this protocol was not registered on ClinicalTrials.gov. Below, we provide an overview of the overall study design, including common approaches and procedures implemented across both phases. Specifically, we review the guiding framework, training and fidelity protocols, as well as data storage and safety procedures. We also present the eligibility criteria used across both phases. Following this, we have separately detailed the protocol and procedures for each phase. See also the CONSORT (Consolidated Standards of Reporting Trials)-EHEALTH checklist in Multimedia Appendix 1 [59].

### Guiding Framework for Intervention Adaptation

#### Innovative Methodological Framework for Intervention Development

This study adopts a community-engaged research (CEnR) approach that integrates the Scrum Agile framework and the multiphase optimization strategy (MOST), combining methods from social science, intervention science, and software development into a single innovative approach to enhance this app-based intervention.

#### Community-Engaged Research

CEnR is a research approach that is particularly valuable for addressing health disparities among disadvantaged populations. In this approach, community partners are actively engaged in the research process and treated as equal collaborators throughout. CEnR establishes a partnership between researchers and communities, fostering shared decision-making at all stages of the research. It may involve a community advisory board (CAB) and incorporate feedback from other community members. In this model, the community and researchers work together to jointly explore issues relevant to the community. Such partnerships lead to the development of culturally appropriate measures and community-informed interpretations of study findings.

#### Scrum Agile Framework

Scrum is a form of Agile methodology used to manage multicomponent software and product development projects [60]. The Scrum model proposes that projects progress through a series of sprints. During a sprint planning meeting, the product is described in terms of the desired outcomes (ie, the features to be developed in the upcoming sprint). At the end of the sprint, these features are tested and integrated into the evolving product.

#### Multiphase Optimization Strategy

We approach the optimization of our JITAI EMI using the MOST framework, which involves 3 phases: preparation, optimization, and evaluation. Activities in the preparation phase include selecting components (eg, content, design features) that are candidates for inclusion in the intervention, and developing a conceptual model. Pilot testing is an important feature of this stage, including an initial proof-of-concept pilot, an open-phase pilot, and a pilot to test the feasibility of conducting a larger optimization trial. In the optimization phase of MOST, steps are taken to refine the intervention to maximize its effectiveness. This is typically achieved through a factorial optimization trial, a fractional factorial trial, a micro-randomized trial, or a combination of these methods. The MOST framework also operates on the principle of continual optimization. In the evaluation phase, the effectiveness of the optimized intervention is assessed via a standard RCT [61]. This study focused on the first and second phases of MOST (ie, preparation and optimization).

### Eligibility Criteria for Phases 1 and 2

#### Participant Eligibility and Screening

Eligibility criteria were largely consistent across phases 1 and 2. Screening was completed using the participant’s own computer or mobile device, with the survey programmed in Qualtrics (Qualtrics International Inc.). The purpose of the assessment was to collect data on self-reported demographics, health status, health behaviors, mental health, and other relevant factors to determine preliminary eligibility for the study.

#### Inclusion Criteria

To be eligible, individuals must (1) be at least 18 years old; (2) identify as a gay, bisexual, or sexual minority man; (3) be HIV positive; (4) have daily access to the internet via a smartphone; and (5) provide informed consent. Based on the study team’s expertise and the existing literature on stimulant use among SMM, inclusion is limited to SMM-LWH reporting stimulant use. Specifically, individuals must report at least five days of stimulant use over a 3-month period within the past 12 months. Participants will be excluded if they report using prescription stimulants only (eg, Adderall).

#### Exclusion Criteria

Participants were excluded if they (1) were unable to complete surveys in English or (2) were currently enrolled in substance use treatment or an intervention study for HIV risk or drug use. The protocol required that any participant who was intoxicated or otherwise unable to provide informed consent be asked to reschedule. In phase 1, we administered the Mini-Mental Status Examination to exclude individuals showing evidence of gross cognitive impairment (ie, score <20), as such issues could compromise their ability to consent and actively participate in the study. Based on participant feedback and consultation with our investigative team, the Mini-Mental Status Examination was omitted in phase 2.

### Data Collection and Measurement

Data collection measures were harmonized across phases 1 and 2. For this study, data were collected from 4 primary sources: (1) online screeners and contact surveys, (2) self-reported online surveys, (3) EMA and EMI, and (4) biometric watch data.

### Harmonized Measures

Baseline and follow-up measures were harmonized across phases 1 and 2. Quantitative outcome measures for feasibility and acceptability included the System Usability Scale [62], Mobile App Rating Scale [63,64], Modified Computer Self-Efficacy Scale [65], Client Satisfaction Questionnaire-8 [66], and eHealth Acceptability Scale [67]. Additional key measures are outlined in Tables 1 and 2 [68-90].

**Table 1:** Measures for the baseline and follow-up assessments (between-person measures).

Variable and between-person measures			Baseline		Follow-up			
**Demographic**								
	Age, race/ethnicity, employment, education, income, substance use, relationship status, sexual orientation, and gender	✓		N/A^a^				
**Stress**								
	The Perceived Stress Scale [68]	✓		✓				
**HIV-specific stress**								
	The HIV/AIDS Stress Scale [69]	✓		✓				
	Everyday Discrimination Scale (HIV) [70]	✓		✓				
	The HIV Stigma Scale 12-item [71]	✓		✓				
**Racial/sexual minority stress**								
	Internalized Homophobia Scale [72,73]	✓		✓				
	Everyday Discrimination Scale (sexual orientation, race) [70]	✓		✓				
**Mindfulness**								
	Five Facet Mindfulness Questionnaire [74]	✓		✓				
	The Toronto Mindfulness Scale [75]	✓		✓				
	Cognitive and Affective Mindfulness Scale Revised [76]	✓		✓				
**Self-compassion**								
	Self-Compassion Scale Short-Form [77]	✓		✓				
**Gratitude**								
	The Gratitude Questionnaire-6 [78]	✓		✓				
**Affect**								
	Positive and Negative Affect Schedule [79]	✓		✓				
**Substance use**								
	Drug Abuse Screening Test (DAST) [80]	✓		✓				
	Shortened Inventory of Problems-Alcohol and Drugs [81]	✓		✓				
**Depression**								
	Center for Epidemiologic Studies Depression Scale [82]	✓		✓				
**Adherence**								
	Simplified Medication Adherence Questionnaire [83]	✓		✓				
**Acceptability and feasibility**								
	System Usability Scale [86]	N/A		✓				
	Mobile App Rating Scale [88]	N/A		✓				

^a^N/A: not applicable (ie, the measure was not administered at that time point).

**Table 2:** Measures for the baseline and follow-up assessments (within-person measures).

Variable and within-person EMA^a^ measures		Daily EMA
**Stress**		
	EMA-Adapted Perceived Stress Scale [84]	✓
	Modified HIV Stigma Scale for EMA [85]	✓
	Measures of daily intersectional stigma [86]	✓
	Daily discrimination related to HIV, SGM^b^ identity, and race/ethnicity [87-89]	✓
**Affect**		
	Alternative Momentary Affect Measure [90]	✓
**HIV TRB^c^**		
	Medication adherence, previous day substance use, and sexual behavior	✓

^a^EMA: ecological momentary assessment.

^b^SGM: sexual and gender minority.

^c^TRB: transmission risk behavior.

### Data Collection Tools

In both phases, screener data, contact information, and quantitative survey data were collected using the Qualtrics survey platform. Quantitative survey data were collected at 2 time points: baseline and 3-month follow-up. Our app is powered by MetricWire (Metricwire Inc), and participants used the app for 90 days, providing both EMA and EMI data via this platform. MetricWire allows the research team to download self-reported EMA data, as well as JITAI EMI engagement data (eg, intervention activity completion). In phase 2, half of the participants received a Fitbit (Google LLC), which was linked to MetricWire. Biometric inter- and intraday data were provided to MetricWire—and thus made available to the study team—via the Fitbit Research API. Qualitative interview data were also collected across both phases. In phase 1, qualitative data consisted of 3 focus groups, and in phase 2, a semistructured follow-up interview; both were audio-recorded via Zoom (Zoom Communications, Inc) and transcribed.

### Ethical Considerations

#### Ethics Approval Statement

All procedures were reviewed and approved by the institutional review boards (IRBs) at the participating institutions: Rutgers University and New York-Presbyterian at Weill Cornell Medical Center. Specifically, Rutgers University executed a single-study IRB authorization agreement with Weill Cornell Medical College (IRB registration number: IORG0000357; protocol number: Pro2021002075).

#### Training, Fidelity, and Protection of Human Participants

Study staff were trained on all relevant procedures for this protocol and provided with a detailed study protocol guide, including (1) confidentiality and general guidelines for participant contact; (2) screening and scheduling protocols; (3) participant contact and tracking databases in Research Electronic Data Capture (REDCap; Vanderbilt University); (4) eligibility check and consent protocols; (5) baseline and follow-up assessment procedures; (6) semistructured interviewing; (7) data security and storage; (8) transcription procedures for qualitative interviews; and (9) monitoring the study email account, Zoom, SMS, and MetricWire instant messenger chat for participant questions.

#### Informed Consent

Before providing consent, all participants were informed about the study procedures, their assigned intervention condition, and the potential risks and benefits of participation. They were also informed of the protections in place to ensure that their data remained confidential and secure, and that their personal information would be protected both during the study and after its completion.

#### Compensation

Participants were compensated for their time and effort in the study. Monetary compensation for both phases was provided via Tango Card Rewards, which allowed participants to redeem their rewards as e-gift cards to various stores and websites. Through Tango Card Rewards, participants also had the option of receiving payment via an e-Visa card or a physical Visa card. In phase 1, participants could earn up to US $315 for completing their baseline assessment (US $50), 3 focus groups (US $50 each), 90 days of EMA (US $90, or US $1 per EMA), and a brief follow-up survey (US $25). In phase 2, participants could earn up to US $180 for completing their baseline assessment (US $50), 90 days of EMA (US $90, or US $1 per EMA), and the follow-up survey and qualitative interview (US $40).

#### Data Protection, Privacy, and Confidentiality

Identifiable information was collected via a contact form in Qualtrics for scheduling assessments, ensuring unique and valid responses, and compensating participants. As a data security measure, contact information was collected separately from the screener data. Specifically, the screener generates a unique participant ID. Once a participant completes the screener, eligible participants—or ineligible participants who opt to share their information for future study opportunities—are forwarded to a new Qualtrics form to provide their contact information. The contact form contains only the participant ID as embedded data. This unique identification number is the sole link between participant contact information and study data. As such, screener data are not stored with participant contact information on the Qualtrics server, nor are they ever downloaded as a dataset. Contact information is stored only in a dedicated contact database designed for scheduling participants, which is powered by REDCap. The study team uses a separate REDCap database to track participant activity (eg, consent completion, appointments, randomization condition, and compensation), which does not include participant contact information. All physical and electronic participant data were free of personal identifiers, with the exception of the unique identification number. Both Qualtrics and REDCap comply with HIPAA (Health Insurance Portability and Accountability Act) standards for privacy protection and use secure sockets layer encryption for all data communications.

Quantitative EMA and EMI survey data are stored on MetricWire’s secure and password-protected platform. While this platform is secure, HIPAA-compliant, and uses encrypted data transmission and storage, we have taken additional precautions to ensure participant confidentiality. When participants are onboarded to the app, they are provided with an anonymous MetricWire email to log-in. Instead of using participants’ names, we use their unique participant ID. As such, no participants’ identifying information is provided to MetricWire, and all data stored on their platform are free of personal identifiers. Biometric data are collected via the Fitbit Sense 2 smartwatch and accessed by the study team through the MetricWire platform. To ensure data security, the study team creates a unique, anonymous university email account for each participant in the watch condition. This email account is encrypted and used to set up the participant’s Fitbit account. Participants’ names are not entered during account creation; only their age, weight, and height are provided to validate biometric data. Participants are then given the study email and password to log-in to the Fitbit app. Once the account is set up, MetricWire allows linking the participant’s Fitbit account, providing access to deidentified biometric datasets.

Qualitative interviews were recorded using the study team’s Zoom Business account, which employs secure sockets layer encryption and is HIPAA-compliant. Recordings were saved as audio only (without video) on a password-protected computer. They were then transferred to a secure university server and deleted from the local device. Recordings were saved using only the unique participant ID as an identifier, and audio files will be destroyed after quality assurance of the transcriptions is completed.

### Phase 1 Procedures

#### Community Advisory Board

A CAB was assembled before participant recruitment for phase 1. Members were recruited from existing research CABs affiliated with 3 universities. Each CAB contributed specific expertise relevant to this study. The first CAB recruited was affiliated with the HIV Prevention Trials Network and comprised individuals LWH. The second CAB recruited was affiliated with the Adolescent Trials Network and included members of the SMM community as well as professionals working in HIV and SMM health services. Additionally, we recruited several members from an existing research CAB of stimulant-using SMM-LWH, affiliated with the START study (R01DA049843).

#### Recruitment and Enrollment (Phase 1)

In phase 1, recruitment, consent, baseline appointments and assessments, as well as all focus group meetings, were conducted online. An initial recruitment email was sent to past or ineligible participants from the investigative team’s contact list who had consented to be contacted for future studies within the past 24 months. The email directed potential participants to our study, providing a link to a brief initial screening survey and the study’s contact information. Once participants expressed interest—either by contacting us or by completing the screening survey—a study team member reached out via phone or email to schedule their baseline appointment. This approach has successfully recruited diverse samples of SMM-LWH in our previous pilot and large-scale studies. At the beginning of the baseline appointment, potential participants were provided with study information and underwent further eligibility screening. Specifically, participants’ age was confirmed by presenting a valid form of identification (eg, driver’s license, state-issued ID, passport, or birth certificate). Participants’ positive HIV status was also confirmed through proof of an antiretroviral therapy prescription or HIV diagnosis (eg, laboratory results, prescription, or medication bottle). If deemed eligible, participants completed an online informed consent form.

#### Study Setting and Timeline (Phase 1)

##### Overview

In phase 1, all study appointments were conducted online via Zoom using the Rutgers Zoom service account. Participants completed a baseline appointment, followed by a 90-day EMI period, which included 3 focus group meetings spaced every 30 days. Participation lasted approximately 3 months. Participants received monetary compensation via e-gift cards through Tango Card Rewards for completing assessments and other study procedures, consistent with previous research. To minimize potential coercion, participants were not compensated for completing any intervention activities. This approach was intended to prevent participants from engaging in the intervention primarily for immediate monetary gain.

##### Baseline Assessment

During the baseline appointment, study staff conducted final eligibility screening and obtained informed consent. Participants were then introduced to the 7 modules forming the basis of the EMI through a review guide. They subsequently completed a brief (1-2 minute) guided mindfulness activity. At the end of the appointment, participants were provided a link to the baseline assessment to complete on their own device and were informed that they would be contacted once the 90-day EMI period was scheduled to begin. All final eligibility screening, informed consent procedures, and the baseline survey were programmed in Qualtrics and completed online. Participants received US $50 for completing the baseline appointment and assessment.

##### Open-Phase Pilot

The 90-day EMI period began once all 10 participants were recruited and had completed their baseline appointments (approximately 1.5 months after the start of recruitment). This timing ensured that the 90 days of EMI and focus group meetings occurred on the same schedule for all participants, which is important for adhering to the Scrum Agile methodology. One week before the EMI period, participants were provided with a guide via email for downloading the app-based EMI. The guide included step-by-step instructions for installing the app on their personal mobile device, information on completing EMA surveys (eg, notifications, expiration, compensation), and guidance on completing a practice survey. Participants were also shown how to use and access each app feature, including on-demand (user-initiated) content, the resources section, and the in-app messenger. The participants’ EMI periods were programmed to begin on the same date. During the 90-day intervention, EMA surveys were delivered once daily and included measures of stress, health behaviors, and mental health. Surveys were completed electronically via the MetricWire mobile app on each participant’s personal smartphone. Participants received US $1 for each completed EMA survey, for a total of up to US $90 if all assessments were completed over the 90-day EMI period.

##### Focus Groups and CAB Meetings

After every 30 days of app engagement, participants were scheduled for a focus group meeting conducted via Zoom (audio-only). A total of 3 focus groups were held. Focus groups were led by a CAB moderator and at least one study team member, who elicited participant feedback using a qualitative focus group guide, starting with broad, open-ended questions and followed by more specific prompts. Specifically, participants reviewed the major components of the Tea Time intervention: (1) the on-demand materials, (2) the intervention activities, (3) the daily positive messages, and (4) the app design. A copy of the focus group guide is provided in Multimedia Appendix 2. In preparation for the focus groups, the research team and CAB reviewed the intervention content and prepared materials for the sessions. This included presenting participants with the intervention content as currently programmed, including screenshots of its presentation within the app and live demonstrations of various app features. Additionally, the app delivers a bank of quotes and images as a “daily positive message,” which are randomly selected. These images and quotes were chosen by the research team during the proof-of-concept pilot. The CAB and research team collaborated to select new images and quotes, in addition to those currently used in the app, and presented them to the focus group for feedback. Focus groups were audio-recorded and transcribed. Participants were compensated US $50 for each focus group, for a total of US $150.

##### Sprints and Iterative Adaptation

**Figure 2:**
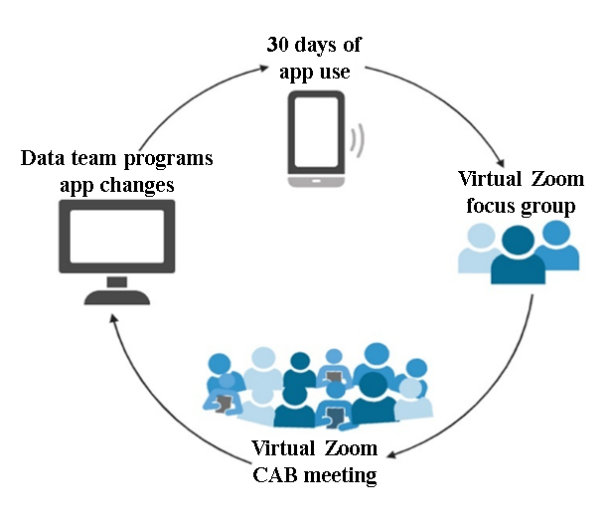
Flow for sprints and iterative adaptation in phase 1 of the study protocol. CAB: community advisory board.

As feedback was gathered, our team used iterative “sprints” following each focus group and CAB meeting to tailor and further optimize the JITAI EMI (see Figure 2). After each sprint, participants continued using the app to review updates in this cyclical approach. In accordance with Scrum Agile methodology, sprints were timeboxed to no longer than 1 month. Three planned sprints are presented in the phase 1 timeline. Consistent with Scrum methodology, a planning meeting was held at the start of each sprint, during which team members created a sprint backlog—a list of tasks to be completed during the sprint. Throughout each sprint, the Scrum team (ie, the data team) took a small set of features from concept to coded and tested functionality. At the end of each sprint, the team conducted a sprint review, during which new functionality was demonstrated to the research team and CAB, who provided feedback. Features were then tested and integrated into the evolving product.

##### Follow-Up Assessment

Participants completed a follow-up assessment online via Qualtrics and received US $25 for their participation. The follow-up assessment included all measures from the baseline assessment, as well as additional measures to assess the acceptability and feasibility of the JITAI EMI.

#### Usability Testing Procedures

**Figure 3:**
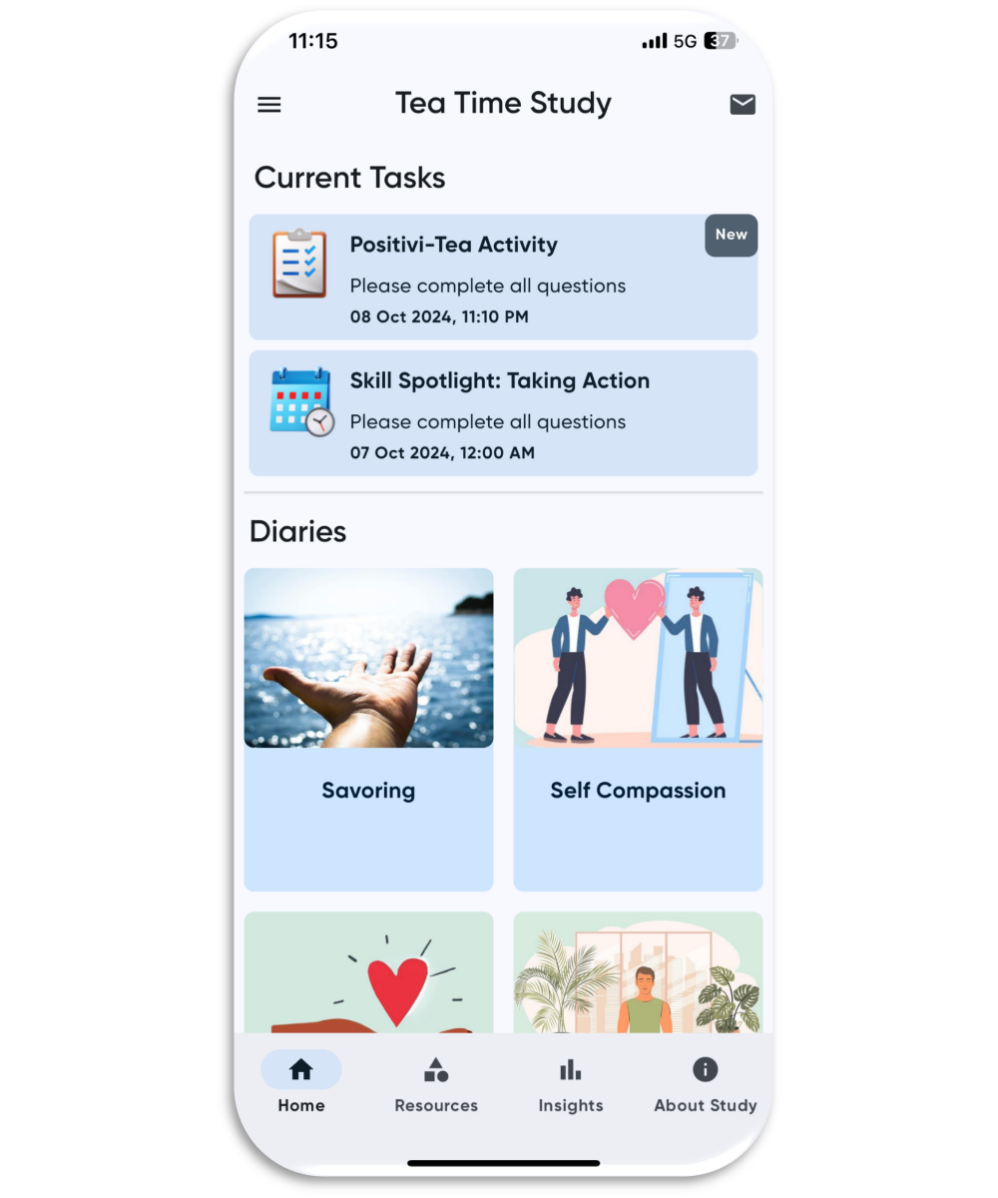
Screenshot of the finalized app, before launching phase 2.

Following the completion of the open-phase pilot, we conducted usability testing. The data team first prepared the app for alpha testing, incorporating final changes and publishing updates so they were accessible to front-end users. Together with our CAB, we conducted usability testing to ensure the app functioned without errors and that data downloads accurately reflected our JITAI design choices (eg, micro-randomization, fixed-interval sampling). We piloted the app for 30 days, providing daily feedback to the data team as programming errors were identified. The data team addressed errors in real time, and the testing team confirmed their resolution. This 30-day period involved iterative development and refinement to finalize all changes to the app. Consistent with Scrum methodology, the team held weekly data meetings to review progress and set action items for the week. See Figure 3 for a screenshot of the app, which was finalized before the launch of phase 2.

#### Preparation Procedures for Factorial Optimization Trial

In preparation for phase 2, we piloted study-related procedures for the factorial optimization trial. First, the data team developed all necessary data tools for the trial. This development occurred alongside usability testing and included creating a screening, contact, and tracking database in REDCap, programming the study appointment dashboard in Qualtrics, and programming the surveys required for the baseline and follow-up assessments. As part of the pilot procedures, research team members tested these databases and trained research assistants (RAs) on their use. The data team also programmed the randomizer in Qualtrics. To prepare for randomization of conditions, a second version of the JITAI EMI was developed to include random craving prompts, which only half of the participants would be assigned to receive. The research team tested all data tools, including the randomizer, to ensure that skip logic and validation functioned correctly. Three months were allotted for the development and testing of these data tools. Once finalized, the study investigators coordinated mock appointments with research staff to run through each condition. As part of this training, RAs practiced the baseline appointment protocol for all 4 conditions.

### Phase 2 Procedures

#### Assessing Feasibility and Acceptability in Phase 2

In phase 2, we recruited and randomized 80 SUSMM-LWH into 1 of 4 conditions: EMI (n=20), EMI + random craving prompts (n=20), EMI with smartwatch (n=20), and EMI with smartwatch + random craving prompts (n=20). We will examine the acceptability and feasibility of adding each of these components, individually and in combination, to our JITAI EMI. Additionally, we will assess the feasibility of enrolling and retaining eligible participants in a factorial optimization trial. Finally, we are establishing proof of concept for collecting passive biometric data via smartwatches.

#### Recruitment and Enrollment (Phase 2)

Recruitment was conducted through both online and in-person methods (eg, provider referrals, email, social media, phone, flyers, and contact databases). All recruitment methods provided participants with a link or QR code to the brief initial screening survey or study team contact information. Participants were recruited via provider referrals, email outreach, flyers, snowball sampling, existing study contact databases, and social media ads. Referrals for participant recruitment were provided by the co-investigator. This involved reaching out to on-site providers with our informational flyer to share with potentially eligible patients, providing direct patient referrals, and placing flyers in departmental waiting areas. We also contacted past or ineligible participants from prior studies who had consented to being contacted for future research via email outreach. Flyers were additionally distributed passively through community collaborators, on-site organizations, and online listservs and mailing lists. For snowball sampling, enrolled participants were provided with a study flyer or QR code for the screening survey to share with potentially eligible individuals in their network.

#### Study Setting and Timeline (Phase 2)

##### Overview

The study timeline included a baseline appointment, a 90-day EMI period, and a follow-up appointment. A final eligibility check and informed consent were conducted at the start of the baseline appointment. Participation lasted approximately 3 months. Study appointments were conducted both online via Zoom and on-site at New York-Presbyterian at Weill Cornell Medicine in a private conference room reserved by the study team. The 90-day EMI period was completed remotely via the Catalyst by MetricWire mobile app on each participant’s personal smartphone.

##### Randomization and Experimental Conditions

After completing the eligibility check, participants were randomized 1:1:1:1 into 1 of 4 conditions: JITAI EMI with random craving prompts, JITAI EMI with smartwatch integration, JITAI EMI with both random craving prompts and smartwatch integration, or JITAI EMI only. This followed a 2×2 factorial design. In all conditions, participants were asked to complete a 90-day EMA, and the JITAI EMI–only group served as the “control.” Randomization assignments were computer-generated via Qualtrics and monitored by the data team.

##### Baseline Assessment

During the baseline appointment, we conducted the final eligibility screening and informed consent procedures. Participants were randomized to 1 of 4 study conditions. For all participants, we reviewed the 7 modules on which the EMI is based, and they completed a brief mindfulness activity. We also reviewed all app features (eg, user-initiated content on the home page, skill spotlight, positive messages, EMA surveys, resources section, in-app messenger), guided participants through downloading the app, and helped them complete a practice survey. Participants then completed the baseline assessment survey online via Qualtrics.

##### Smartwatch Assignment

Participants randomized to a watch condition received a Fitbit Sense 2 smartwatch during the baseline assessment, which was used for passive biometric data collection. Integration of the smartwatch with the MetricWire app was essential to pair EMA and biometric data; therefore, participants were provided with clear instructions on this process. The Fitbit Sense 2 can integrate with the MetricWire mobile app, allowing participants’ passive biometric data to be linked with real-time, individual-level EMA data. The Fitbit collects continuous data on physiological stress reactivity, heart rate variability, heart rate, skin temperature, and blood oxygen saturation. Fitbit also provides API access, allowing MetricWire to regularly download intraday data from their server without requiring participants to visit the study site. In a recent review of smartwatch brands used in research, Fitbit was used in twice as many validation studies and was registered in ClinicalTrials.gov studies 10 times as often as other brands.

##### EMA/EMI 90-Day Period

Participants completed a 90-day EMA/EMI period, which was introduced during the baseline appointment and scheduled to begin the following day. The EMA and EMI were delivered via the Catalyst by MetricWire app, a leading platform for EMA data collection and JITAI EMI delivery. MetricWire offers several sensor capabilities that enable passive data collection, including smartwatch integration, as described above. While participants were compensated US $1 for each daily EMA survey, there was no monetary incentive for viewing any other app-delivered prompts, including intervention content or activities.

##### EMA Design Details: Planned Missing Design

Participant feedback from our proof-of-concept pilot, Positively Healthy, as well as phase 1 of this study, informed our decision to explore strategies to reduce burden and optimize EMA design. This led us to implement a Planned Missing EMA design in phase 2. A Planned Missing EMA design can reduce repetition, which in turn is expected to lower participant burden [91,92]. A Planned Missing EMA design is one in which items are intentionally omitted on alternating days. This approach not only reduces redundancy and participant burden but also allows researchers to maximize data collection and use validated scales. Essentially, this design provides participants with different survey items each day. The variety in survey items is expected to improve attention and engagement while reducing boredom and response fatigue, all of which can contribute to careless responding [93]. Planned Missing designs are also associated with greater quantity and quality of participant responses [94] and may reduce measurement error while improving variance in EMA data. Although this design has become increasingly popular in longitudinal research, limited studies have examined its utility in EMA [92,94,95]. In phase 2, all participants received a Planned Missing EMA design in which each item was asked two-thirds of the time, ensuring that each item had 67% complete data. This results in each pair of items for a given construct having 33% complete data for covariance estimates, which is standard for a simple matrix design [92].

##### EMA Design Details: Fixed Interval Schedule

Feedback from the proof-of-concept pilot also informed our decision to use a fixed-interval EMA design. During the 90 days following the baseline appointment, all participants received a daily notification to complete the EMA, which was delivered on a fixed-interval schedule. Specifically, all participants are asked to complete 1 EMA per day (approximately 5 minutes), which is delivered at 6 PM and expires at midnight, providing a 6-hour completion window. A reminder is sent halfway between the EMA’s delivery and expiration if the survey has not yet been completed. In the EMA, participants report their current mood, substance use cravings, stress, and other health behaviors. A 24-hour recall is used to assess substance use and medication adherence from the previous day. Participants in the “random craving prompt” condition also received single-item EMA craving prompts 3 times per day, in addition to the once-daily evening EMA survey. These single-item craving prompts were delivered randomly within 3 time windows: 8-10 AM, 2-4 PM, and 8-10 PM, each with a 2-hour expiration window. To minimize participant burden from excessive notifications, no reminders were sent for the random craving prompts.

##### JITAI EMI Design Details

If participants reported stress or cravings in the EMA, they were micro-randomized to receive a JITAI activity (*P*=.70). The selection of a specific JITAI activity occurred through a further micro-randomization process. Once a participant was randomized to receive a JITAI activity, they were next randomized to 1 of 8 skills (*P*=.12), and then the JITAI randomly selected 1 activity from a bank of 4 possible activities (*P*=.25), each designed to help the participant practice the assigned skill.

##### Follow-Up Assessment

Participants completed a follow-up assessment after the 90-day EMA/EMI period. This assessment included an online survey and a semistructured qualitative interview (30-40 minutes) conducted by the RA. Interviews were conducted either in person at the Weill Cornell Medicine site or virtually using the study team’s Rutgers single sign-on–secure Zoom account. All interviews were audio-recorded, either using a voice recorder or via Zoom (audio only). All participants were asked general questions regarding the acceptability of their treatment condition. Additionally, questions were tailored based on each participant’s assigned condition (eg, only participants in the smartwatch conditions were asked questions relevant to that condition). A copy of the follow-up interview guide is provided in Multimedia Appendix 3. Upon completion, participants received US $40 for the follow-up assessment, in addition to compensation for any completed EMAs over the study period (US $1 per day).

#### Planned Analyses

In both phases of the study, we plan to conduct qualitative and quantitative analyses to assess the acceptability and feasibility of our JITAI EMI. Planned analyses will also evaluate the feasibility of the intervention design, specifically testing the implementation of an MRT embedded within a 2×2 factorial optimization trial.

#### Qualitative Analyses

Qualitative data will be analyzed using qualitative content analysis [96]. Sections of the interviews related to the acceptability of each condition, program satisfaction, and perceived relevance of program content will be coded separately. Feasibility will be assessed by coding participant responses to questions regarding barriers and facilitators to app engagement. Specifically, we will begin coding by first identifying indicators within participant responses. Similar indicators that emerge across participants will be grouped together and assigned tentative labels [97]. Consistent with qualitative methodology, written coding notes and memos will be maintained throughout the analysis. This process will condense participant data into a smaller set of themes [97]. Interrater reliability will be assessed using Cohen κ between 2 coders. Methodological triangulation will be used to compare quantitative and qualitative findings, further validating the acceptability and feasibility of the intervention.

#### Quantitative Analyses

We will first assess the feasibility of recruiting and randomizing participants into the 4 study conditions. To do so, we will develop a CONSORT diagram illustrating the enrollment cascade. Target benchmarks include more than 70% of eligible participants providing consent and over 60% of consented participants being successfully randomized [98]. We will also conduct attrition analyses to examine the proportion of randomized participants who drop out before initiating the intervention, as well as those who discontinue during the 90-day intervention period. Our target benchmark (>80%) is based on a review of retention rates in other clinical trials [99,100]. We will conduct randomization checks and examine between-group differences in attrition to ensure no significant differences exist by condition in variables such as race/ethnicity, education, income, or relationship status. Chi-square tests will be used to examine the proportion of participants in each condition who are retained through the postintervention assessment. Feasibility will also be assessed by examining adherence to each treatment condition.

For participants in the smartwatch conditions, feasibility will be defined by the rate of returned watches after the 90-day period. In previous EMA studies, we observed a very high return rate (74/80, 92%) among SMM-LWH. The target benchmark for this sample of SUSMM-LWH is >80%. We will also assess the feasibility of collecting passive biometric data from the smartwatches. The study will provide insight into the feasibility of collecting continuous stress reactivity data from smartwatches among SUSMM-LWH. Certain factors, such as forgetting to charge the device, may result in nonrandom missing data. We will use Fitbit’s API to access stress reactivity data on their server and regularly download it to examine patterns of missing data.

Feasibility for the EMA portion of the study will be assessed across all conditions by examining the percentage of completed EMA surveys. In our pilot work, participants completed 66.19% of EMAs (median 78.21%), and our target benchmark for this study is a completion rate over 80% [101]. Adherence to the EMI will be defined as the average number of intervention activities participants completed, as well as the average number of views for positive messages and on-demand materials. In our pilot work, participants completed 83.27% of intervention activities (median 94.44%) and viewed 68.9% of daily positive messages (median 82.72%) over the 90-day period. Based on these data, as well as findings from other mobile health (mHealth) studies, our a priori target benchmark for app engagement is over 80% completion for both intervention activities and positive messages. Completion data will also be used to help identify the ideal length of the intervention. Should completion rates for the EMA or intervention activities fall below 80% over the 90-day period, we will determine the ideal intervention duration that balances maximal completion rates (≥80%) with sufficient power to detect an effect. Acceptability will be assessed using several measures. For the EMI, a score over 72.75 on the System Usability Scale is considered the benchmark for “good” or satisfactory usability [62]. Regarding app quality, an average score of 3 or higher on all subscales of the Mobile App Rating Scale is considered acceptable [64].

#### Exploratory/Preliminary Outcome Analyses

Although our primary aims were to assess acceptability and feasibility, we plan to leverage data collected in this study for exploratory and preliminary outcome analyses examining stress, substance use, and adherence. We will use traditional regression-based analyses to assess between-person effects, as well as main and interactive effects of random craving prompts and smartwatch integration on self-report outcomes, in addition to objective measures of substance use and adherence. We will also conduct multilevel analyses to examine within-person effects of JITAI EMI delivery on same-day substance use and adherence using lagged variables. Additionally, passive biometric data will allow us to examine stress reactivity in relation to participants’ responses to random craving prompts and JITAI EMI intervention delivery.

## Results

**Figure 4:**
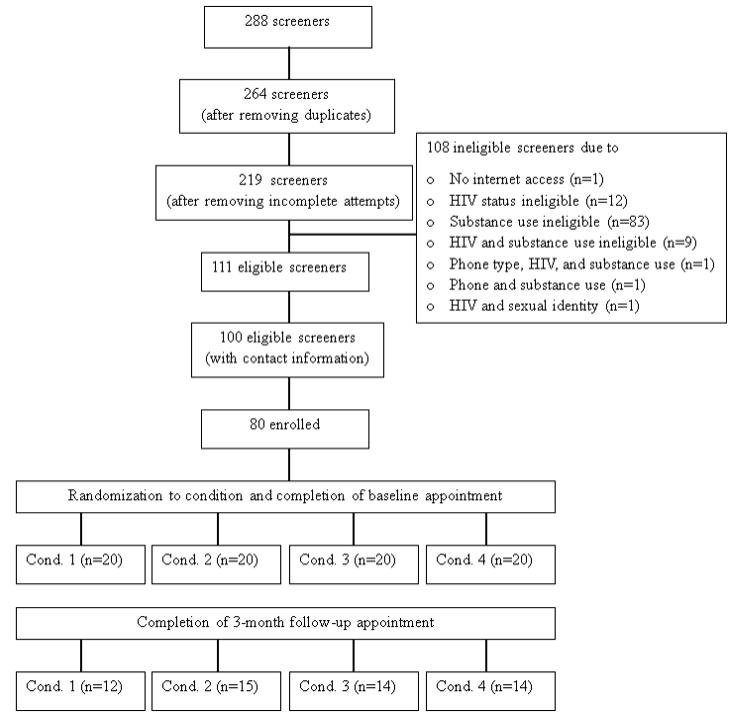
Enrollment cascade and attrition for the phase 2 pilot factorial optimization trial. Condition 1: EMI only; condition 2: EMI + random craving prompts; condition 3: EMI + smartwatch; condition 4: EMI + random craving prompts + smartwatch. EMI: ecological momentary intervention. EMI: ecological momentary intervention.

The project received funding in February 2022. Data collection for phase 1 began in October 2022 and concluded in December 2023. Phase 2 was launched in July 2024, with data collection completed by August 2025. Figure 4 presents the enrollment cascade and attrition results. Data analysis assessing acceptability and feasibility is currently in progress, with initial results expected to be submitted for publication between late 2025 and early 2026.

## Discussion

### Principal Findings

This paper outlines the study protocol for phase 1 and phase 2 of the Tea-Time study. In executing this protocol, we are tailoring our app-based EMI content and optimizing the JITAI design of the EMI. Specifically, in phase 1, we focused on adapting the intervention content to the unique needs and preferences of SUSMM-LWH. To accomplish this, we employed a CEnR approach, working closely with study participants and a CAB to guide the tailoring of our intervention. In addition to tailoring the content, we incorporated participants’ suggestions for improving the system usability of the app. In phase 2, we piloted an MRT embedded within a 2×2 factorial optimization trial. Findings from the MRT will provide valuable insights, enabling further optimization of our JITAI design (eg, decision points, tailoring variables, decision rules). Findings from the randomized factorial trial will provide insights into whether smartwatch integration or random craving prompts are feasible to implement in this intervention. These findings will also clarify whether the combination of these new features is as acceptable to participants compared with either feature alone. Additionally, these data can be leveraged to assess preliminary efficacy for both proximal and distal outcomes.

### Strengths and Limitations

This study takes a CEnR approach, integrating the Scrum Agile framework with MOST, thereby combining methods from social science, intervention science, and software development into a single, innovative approach to enhance this app-based intervention. In particular, our implementation of the Scrum Agile framework alongside MOST for intervention optimization strengthens both the study design and protocol. First, these 2 approaches are highly complementary, as both propose iterative processes for adapting app-based interventions. Additionally, each provides a distinct framework to guide software development and intervention optimization. Scrum, most commonly used in technology-based fields such as computer science, is a framework for managing multicomponent software and product development projects. This framework guides us in efficiently managing data-related tasks as we program adaptations to the JITAI EMI and release different iterations of the app for alpha and beta testing. The MOST approach provides a complementary framework specific to intervention optimization, informing design choices for piloting an optimization trial of our adapted app. In essence, Scrum provides guidance on how to adapt the app, while MOST provides a framework for deciding which features should be adapted.

As part of phase 2, we are piloting the feasibility of conducting an MRT embedded within a factorial optimization trial with SUSMM-LWH. To optimize high-intensity adaptive interventions, an MRT is recommended [61,102]. The MRT is conducted under an n-of-1 framework and functions as a factorial design in which each individual is repeatedly exposed to each intervention condition over time [61]. This approach is useful for optimizing features within the JITAI EMI; however, the optimization of other features requires a standard factorial design. For example, it is not feasible to randomly assign the same participant to wear a smartwatch on some days and not others over a 90-day assessment period. Similarly, because random craving prompts are a feature of the EMA rather than the JITAI EMI, this component is also best evaluated within a standard factorial design. This combined approach to optimization is also a strength of the current protocol and will minimize the need for future optimization trials. As such, it is also cost-effective. Further, by accelerating the optimization process, we come closer to achieving a fully optimized intervention ready for evaluation and implementation. In a future RCT, the EMA design will also allow us to collect day-level data on intervention efficacy.

One potential area of concern is that participant burden [103] and response fatigue [93,104] associated with intensive repeated-measures designs threaten data validity. In addition, burden and fatigue are both associated with poorer EMA compliance [105]. Participant burden is an especially important concern for researchers working with SUSMM-LWH, as this population is already disproportionately impacted by health-related burden and other acute daily stressors. In addition, prior research shows that compliance with EMA is a challenge among substance users more generally [106] and among SUSMM [107]. Some studies have also found that racial and ethnic minority SMM and SUSMM are more likely to show poor compliance with EMA [10,108]. Poor compliance poses a significant concern regarding the feasibility of JITAI EMI delivery with SUSMM-LWH. Given that the success of a JITAI EMI relies heavily on the validity of EMA data as well as compliance with the EMA, it is important to optimize EMA methods and design before scaling up this intervention for SUSMM-LWH.

Improving EMA compliance is a vital first step in optimizing this JITAI EMI. Compliance can be improved by reducing participant burden [105]. One approach to minimize burden is to integrate ambulatory assessment into the EMA/EMI, which would facilitate passive data collection [109-111]. Smartwatches can be used to collect data such as heart rate and physiological stress responses [111], which can then be used to trigger EMI intervention content [110]. Smartwatches can also integrate with mobile apps, allowing participants to receive notifications, surveys, and EMI content directly on their watch, making engagement effortless and accessible. We expect that smartwatch integration for participants in this 2×2 trial will improve both EMA compliance and EMI engagement.

Compliance is also related to design choices [105]. In our proof-of-concept pilot, participants reported often missing their EMA due to its timing (random interval sampling) and expiration. In this protocol, we have addressed this issue by incorporating participant feedback into our EMA scheduling, moving from a random-interval design to a fixed-interval design. A fixed-interval design allows participants to set aside dedicated time to complete the EMA and may improve compliance. Of course, this change introduces certain limitations, as random-interval EMA is believed to provide better ecological validity. This is being addressed in part through our 2×2 design, which tests the integration of random-interval craving prompts in addition to the fixed-interval evening survey. Importantly, random craving prompts will trigger intervention content and will therefore be a key feature for delivering in-the-moment support for SUSMM-LWH. We expect that this combined sampling approach (eg, fixed + random interval) may further optimize our JITAI EMI.

### Conclusions

Ultimately, this project aims to develop a fully optimized EMI that is both culturally relevant and acceptable to our target audience by eliminating sources of community-program mismatch in our intervention content. Additionally, we aim to further optimize the intervention by incorporating 2 additional features that may enhance engagement and effectiveness while minimizing participant burden and other potential barriers to engagement. If we are successful in these aims, this app-based JITAI EMI will offer a cost-effective intervention approach that provides SUSMM-LWH support in real time, in their daily lives, when it is most needed.

## Data Availability

The data that support the findings of this study are not publicly available due to their sensitive nature and ethical restrictions concerning participant privacy. The data are available upon reasonable request, following a review process to ensure participant confidentiality. Data requests may be sent to the first author (KMS).

## Appendix

Multimedia Appendix 1 CONSORT (Consolidated Standards of Reporting Trials)-EHEALTH checklist.

Multimedia Appendix 2 Focus group guide for phase 1.

Multimedia Appendix 3 Qualitative interview guide for phase 2.

Multimedia Appendix 4 Peer review report by the HIBI -HIV/AIDS Intra- and Inter-personal Determinants and Behavioral Interventions Study Section (National Institutes of Health, USA).

## Abbreviations

CAB: community advisory board
CEnR: community-engaged research
CONSORT: Consolidated Standards of Reporting Trials
EMA: ecological momentary assessment
EMI: ecological momentary intervention
HIPAA: Health Insurance Portability and Accountability Act
IRB: institutional review board
JITAI: just-in-time adaptive intervention
LWH: living with HIV
mHealth: mobile health
MOST: multiphase optimization strategy
MRT: micro-randomized trial
RA: research assistant
RCT: randomized controlled trial
REDCap: Research Electronic Data Capture
SMM: sexual minority men
SU: substance using
TRB: transmission risk behavior
