# Supplementary material for: Optimizing an App-Based Just-in-Time Adaptive Intervention for Stimulant Use Among Sexual Minority Men Living with HIV: Protocol for a Community-Engaged Research Approach and Hybrid-Experimental Design
Source: JMIR Res Protoc. 2025 Dec 2;14:e76741. doi: 10.2196/76741 (PMC12709163; doi:10.2196/76741)
Supplement: Multimedia Appendix 2 [file resprot_v14i1e76741_app2.pdf]

Focus Group Interview Guide

The exact wording of the questions is to be determined and developed in collaboration with our community advisory board throughout the activities of the grant.

General Interest in Positive Psychological Programs

Conception of happiness, psychological well-being, and life satisfaction

1. When someone says that they are feeling happy, what does this mean?
    a. How would you define "happiness"?

2. What are the ingredients for achieving happiness at home, at work, etc.?

3. What would a "very satisfying" life look like?

Barriers for achieving well-being

1. What are some barriers to life satisfaction that may decrease life happiness?
2. What are some life "stressors" or "pressures" that decrease your life satisfaction or happiness at home or work?

Facilitators/strategies to enhance well-being

1. What are some things you do to help decrease or manage your stress?
2. What strategies do you use to increase your happiness and life satisfaction when faced with stress or pressure?

Receptivity to a positive psychology app or program

1. Would you participate in a program designed to increase happiness, life satisfaction, and psychological well-being?
    a. Why or why not?"

2. What are some life "stressors" or "pressures" that decrease your life satisfaction or happiness at home or work?

RUTGERS | eIRB
APPROVED
IRB ID: Pro2021002075
Approval Date: 12/22/2021
Expiration Date:

Specific Tea Time App Questions

Engagement Questions

1. What is your favorite aspect of the Tea Time mobile app?

2. The app focuses on eight skills: noticing and savoring positive events, gratitude, mindfulness, behavior activation, positive reframing, personal strengths, and acts of kindness. (Note: write skills on board)
   a. Which was your favorite skill?
   b. How did it resonate with you?

Program Length and Content Questions

1. What are your thoughts on the Tea Time app now that you have been part of this program?
   a. What do you think was most helpful about the approach?
   b. What do you think was least helpful about the approach?

2. Did the amount of activities you received over the last 90 days feel like enough?
   a. Do you think you should have received more activities?
   b. Do you think you should have received less?

3. Thinking about the app, what parts of it felt most relevant to your needs?
   a. Probe for thoughts on app or program activities:
      i. Random Acts of Kindness
      ii. Mindfulness
      iii. Noticing and Savoring Positive Events
      iv. Gratitude
      v. Personal Strengths
      vi. Positive Reappraisal
      vii. Behavioral Activation

4. How much do you feel the Tea Time program addressed your needs as an HIV+ GBM?

5. What changes would you recommend to make to this app or program more appealing /more relevant to HIV+ GBM?

   a. Is there anything we could specifically add to make it more relevant for people who use substances or struggle with substance use cravings?

6. What other suggestions do you have for adapting this app? What would you do differently?

RUTGERS | eIRB
APPROVED
IRB ID: Pro2021002075
Approval Date: 12/22/2021
Expiration Date:

Perspectives on Mode of Delivery

1. How did you feel about the app platform?
    a. Do you wish the app had been designed differently?
    b. What did you like about it?

2. What was your favorite feature of this app?
    a. Probe for positive messages, program activities, on demand content….

3. What do you think would be the best mode to deliver this type of program?
    a. Did you like the app as a mode of delivery?
    b. Would you have preferred an in person program?

4. What additional app features would improve the acceptability of this program?

5. About how many program activities do you think you were prompted to participate in?
    a. What percentage of the time did you engage in a prompted activity?
    b. What do you think prevented you from engaging in the program activities?

6. Did you find the on demand material to be helpful?
    a. How often did you utilize it?

7. Did you like the daily positive messages?
    a. How did you feel these impacted your mood each day?

8. If we were going to do this program again, what do you think would be necessary for Tea Time to be successful?
    a. What specific changes would you recommend making to the app or program to allow this to happen?

Participant / Population specific

1. Did you think that the Tea Time app was interesting? Why or why not?
    a. How do you think we can maximize interest and participation on this app in the future?
    b. What obstacles did you encounter to participating?
        i. Emotional obstacles?
        ii. Social barriers?
        iii. Feeling comfortable with the app content?

2. What do you think are the best ways to address these obstacles for HIV+ GBM in the future?

RUTGERS | eIRB
APPROVED
IRB ID: Pro2021002075
Approval Date: 12/22/2021
Expiration Date:

Wrapping Up

1. What other thoughts, reflections, suggestions or comments do you have about the experience of participating in this program?

Thank you so much for taking the time to talk with me today. Your thoughts and opinions about taking part in this program will help us learn about user experiences with Tea Time, and how it could be improved if we do it again. We hope that this study will be helpful for HIV+ GBM in our community and your participation is a part of that. Thanks again for being a part of this and for meeting with me today to share your perspective.

RUTGERS | eIRB
APPROVED
IRB ID: Pro2021002075
Approval Date: 12/22/2021
Expiration Date:
