# Supplementary material for: Optimizing an App-Based Just-in-Time Adaptive Intervention for Stimulant Use Among Sexual Minority Men Living with HIV: Protocol for a Community-Engaged Research Approach and Hybrid-Experimental Design
Source: JMIR Res Protoc. 2025 Dec 2;14:e76741. doi: 10.2196/76741 (PMC12709163; doi:10.2196/76741)
Supplement: Multimedia Appendix 3 [file resprot_v14i1e76741_app3.pdf]

RUTGERS
Institute for Health, Health Care Policy, and Aging Research

Rutgers, The State University of New Jersey
112 Paterson Street
New Brunswick, NJ 08901

www.ihhcpar.rutgers.edu
Phone: 732-932-8413
Fax: 732-932-6872

Follow-Up Appointment : Qualitative Interview Guide

Introduction to Interview

First, thank you again for being part of our study! We really appreciate the time you've taken to test out our app over these past three months. Next, we will do a short interview where I will be asking you to provide your feedback on the app. Your feedback is very important to us and will help us tailor the app after the study. Please feel free to share anything that you did not like about the app! The purpose of this study is for us to understand what worked and more importantly what didn't. Any advice, tips, or feedback on how we can improve the Tea Time app are greatly appreciated.

Before we get started, I just want to remind you that this part of the appointment will be recorded. Only the audio recording for the interview will be saved, however, if it makes your more comfortable, you are welcome to turn off your camera for this part of the appointment.

Next Button
End Page

Acceptability / Engagement Questions

1. What was your favorite aspect of the Tea Time mobile app?

- *Probe here as needed*

2.What was your least favorite aspect of the Tea Time mobile app?

- *Probe here as needed*

3. The app focuses on eight skills: savoring positive events, gratitude, mindfulness, behavior activation, positive reframing, personal strengths, self-compassion, and acts of kindness.

Note: Share Screen on the Next Page

Next Button
End Page

RUTGERS
Institute for Health, Health Care Policy, and Aging Research

Rutgers, The State University of New Jersey
112 Paterson Street
New Brunswick, NJ 08901

www.ihhcpar.rutgers.edu
Phone: 732-932-8413
Fax: 732-932-6872

4. Which was your favorite skill and how did it resonate with you?

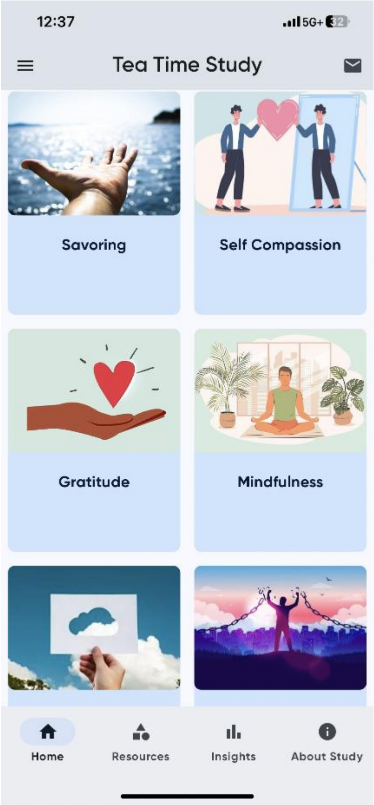

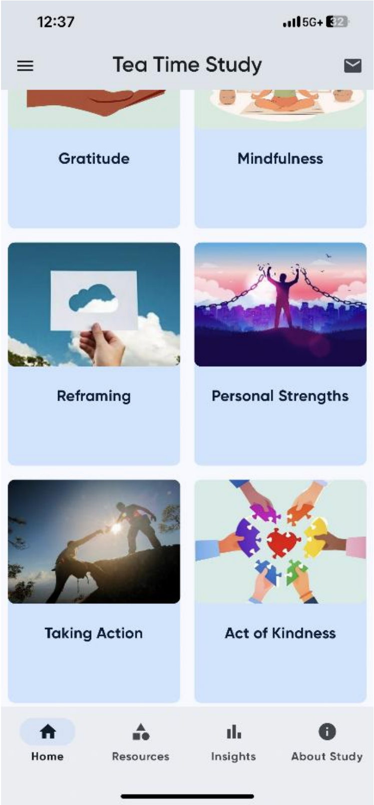

Next Button

Institute for Health, Health Care Policy, and Aging Research
Rutgers, The State University of New Jersey
112 Paterson Street
New Brunswick, NJ 08901
www.ihhcpar.rutgers.edu
Phone: 732-932-8413
Fax: 732-932-6872

5. Which was your least favorite skill? Why?

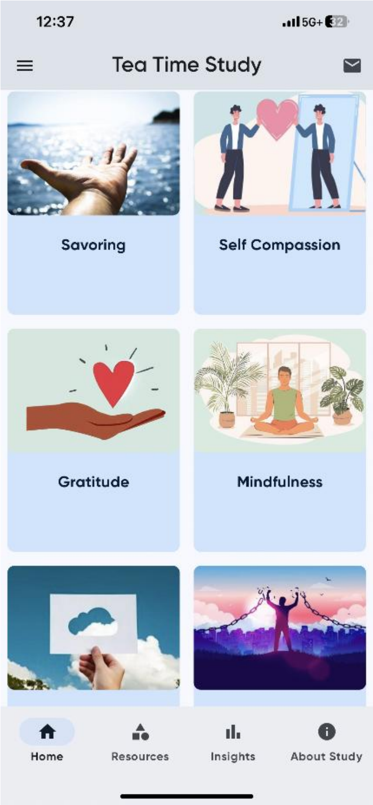

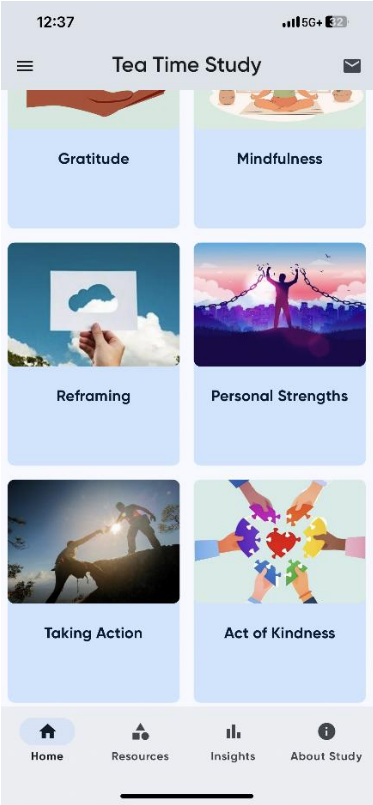

(Stop screen share before moving on)

Next Button
End Page

Perspectives on Mode of Delivery

6. How did you feel about the app platform?
- Do you wish the app had been designed differently?
- What did you like about it?

7. The app included several features, the home screen, resources, insights, and in-app messenger.

- Which features of the app did you find to be most useful?
- *Probe here as needed*

Note: Share Screen on the Next Page

Next Button
End Page

Rutgers
Institute for Health, Health Care Policy, and Aging Research

Rutgers, The State University of New Jersey
112 Paterson Street
New Brunswick, NJ 08901

www.ihhcpar.rutgers.edu
Phone: 732-932-8413
Fax: 732-932-6872

8. The home screen offered several features: Skill Spotlight, Daily Evening Survey, Daily Dose of Positivi-tea!, and Diaries (aka. The 8 Skill Modules).
   - Which of these was your favorite feature on the home screen?
   - Why?

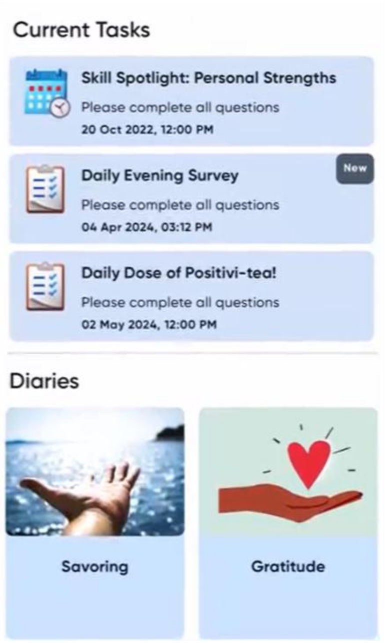

(Stop screen share before moving on)

Next Button
End Page

9. Skill-Spotlight was a new feature we added based on prior study participant's feedback. This rotated weekly, highlighting a new skill each week. Skill Spotlights also provided 3 new activities each week.
   - What did you think of this feature?
   - Did you participate in any of the Skill Spotlight activities?
     - Were there any that stood out as being really interesting or helpful
   - Do you have any advice or feedback on how we can improve the Skill Spotlight feature of our app?

10. Sometimes after the daily surveys, you received a notification prompting you to do a brief activity.
    - About how many activities do you think you were prompted to do ?
    - What (if anything) do you think prevented you from engaging in the program activities?
    - Do you have any feedback on how they could be improved?
    - Did the number of activities you received over the last 90 days feel like enough?
    - Do you think you should have received more activities?
    - Do you think you should have received less?
    - Overall, what did you think about the quality of these activities?
      i. How did they impact your mood after completing them?

Rutgers, The State University of New Jersey
112 Paterson Street
New Brunswick, NJ 08901

www.ihhcpar.rutgers.edu
Phone: 732-932-8413
Fax: 732-932-6872

11. The "Daily Dose of Positivi-tea!" was a positive message you received twice a day.
    - Did you like these daily positive messages?
        - Why/Why not
    - How did you feel these impacted your mood each day?
    - What did you think of the images used for these, did they feel relevant to you?
    - Do you have feedback on how we could make these more relevant for you?

12. The "8 Skill Modules" under the Diaries section on the home screen included information about each skill, as well as additional activities.
    - Did you find the home screen's "8 Skill Modules" material to be helpful?
        - Why or why not?
    - How often did you utilize it?
    - Do you have any recommendations to make this material more useful or engaging?

13. What additional app features would improve the acceptability of this program?

14. If we were going to do this program again, what do you think would be necessary for the Tea Time app to be successful?
    - What specific changes would you recommend making to the app to allow this to happen?

Relevance to Population

15. Thinking back to the Tea Time app as a whole, which parts felt most relevant to your needs?

16. How do you think we can maximize interest and participation on this app in the future?
    - Did you feel comfortable with the apps content?
    - What obstacles did you encounter to participating?
    - What do you think are the best ways to address these obstacles for sexual minority male (or GBM) living with HIV in the future?

17. How well do you feel the Tea Time app addressed your needs as a sexual minority male (or GBM) living with HIV?

18. What changes would you recommend to make to this app more appealing /more relevant to sexual minority men living with HIV
    - Do you think there is anything we could specifically add to make it more relevant for people who use substances or struggle with substance use cravings?

19. What other suggestions do you have for adapting this app? What would you do differently?

Watch Integration [Only for People in Watch Condition]

20. Prior to this study, did you wear a smart watch or fitness watch (Apple watch, Fitbit, Garmin, etc).?

21. How did you feel about wearing a Fitbit watch for this study?
    - Did you like wearing it? What did you like most about it?
    - Did you experience any challenges to wearing the watch over the past 90 days?
        - *Probe here*

22. Did you receive Tea Time app notifications to your watch?
    - Did you find this helpful?
    - Why or why not?
    - Do you think receiving notifications to your watch made you more likely to engage with the Tea Time app?

23. How else do you think wearing the Fitbit influenced your engagement with the Tea Time app?

24. Are there other ways in which you wish the watch was integrated with the Tea Time app?

25. Do you have any feedback re: the watch aspect of this study?

Combining Web Intervention and App

We are almost done, we have just a few more questions.

So, the Tea Time program has been delivered in a variety of formats, including in person and as a web-based program. Part of this study was to tailor it for app-based delivery.

26. What do you think would be the best mode to deliver this type of program?
    - Did you like the app as a mode of delivery?
    - Would you have preferred an in person program?
    - Would you have preferred a web-based program?

27. We are considering whether a combination of delivery modes might be better. We'd like to get your thoughts on the web-based version of the program. To give you an overview of it, we are going to watch a brief video.

    *Participants will watch a 1.5 min Video with information about the Web Program.

    Note: Share Screen on the Next Page
    Next Button
    End Page

RUTGERS
Institute for Health, Health Care Policy, and Aging Research

Rutgers, The State University of New Jersey
112 Paterson Street
New Brunswick, NJ 08901

www.ihhcpar.rutgers.edu
Phone: 732-932-8413
Fax: 732-932-6872

Embed Video (Note video file is in folder: C: The Tea Team Dropbox\The Tea Team - general folder\R34 Tea Time documents\2.0 TT PHASE 2\Programming\Qualtrics\Follow-Up Interview Guide)

(Stop screen share before moving on)

Next Button
End Page

28. Based on this brief video, what do you think of the web-version of our program?
    - What features do you find most interesting or appealing about this version of the program?
    - Is there anything you dislike about this program?
    - Would you be willing to participate in a study with the web-based version?

29. We think that the app may be a good adjunct to the web-based program. The web-program would be something you engage with once a week for 2 months and the app would provide support in between and for one month after.
    - Do you think that is something you would be interested in?
    - Would you be willing to participate in a study where you use the web-based version for 8 weeks, while also using the app?

Wrapping Up

30. What other thoughts, reflections, suggestions or comments do you have about the experience of participating in this program?

Thank you so much for taking the time to talk with me today. Your thoughts and opinions about taking part in this program will help us learn about user experiences with Tea Time, and how it could be improved if we do it again.

We hope that this study will be helpful for our community and your participation is a part of that. Thanks again for being a part of this and for meeting with me today to share your perspective.
