# Supplementary material for: Optimizing an App-Based Just-in-Time Adaptive Intervention for Stimulant Use Among Sexual Minority Men Living with HIV: Protocol for a Community-Engaged Research Approach and Hybrid-Experimental Design
Source: JMIR Res Protoc. 2025 Dec 2;14:e76741. doi: 10.2196/76741 (PMC12709163; doi:10.2196/76741)
Supplement: Multimedia Appendix 4 [file resprot_v14i1e76741_app4.pdf]

1R34DA053999-01A1 Sizemore, Kayla

RESUME AND SUMMARY OF DISCUSSION: This application proposes to first examine the feasibility and acceptability of a positive affect and mindfulness intervention for substance-using sexual minority men (SUSMM) living with HIV that has been adapted for delivery through a mobile app by reconfiguring the intervention content to be delivered as an Ecological Momentary Intervention. This means that the intervention content is delivered to an individual in response to an ecological momentary assessment which is intended to deliver the intervention content at the time the individual needs it most. The team presented pilot work showing the efficacy of this approach using an in-person version of the intervention. In the second phase of the application, the team will seek to test the acceptability and feasibility of two features that are intended to enhance the delivery of the ecological momentary intervention: random craving prompts delivered throughout the day and the use of smart watch to enhance the ability of the app to deliver the just-in-time intervention content. The proposed development application is building upon a tested positive affect and mindfulness intervention that is now being modified to address a group that is at high risk for HIV transmission - SUSMM living with HIV. The study team is very strong and has the expertise to carry out this complex study. The investigative team has been highly responsive to the concerns that were raised in the prior review of this application. The substance use inclusion criteria, which had been all substance use, was limited to stimulant users which is better aligned with the team's expertise. The addition of a blood draw, hair sample collection and the use of the passive data collected by the smart watches further addressed prior concerns. In addition, the team has addressed a number of other minor concerns. As a result, the committee's overall enthusiasm for this much improved resubmission was very high.

DESCRIPTION (provided by applicant): In the United States (U.S.), sexual minority men (SMM) are disproportionately affected by HIV compared to the general population. For this population, intersectional sexual minority and HIV-related stress add to general life stressors to increase health risks. Research demonstrates a bidirectional association between psychosocial stress and physiological HIV progression, including CD4 decline and increased viral load. However, stress is not only associated with HIV progression, it is also linked to transmission risk behaviors (TRB), such as medication non-adherence and substance use. Substance use is a particularly important risk factor for HIV transmission. In addition to injection drug use risk, recreational use is associated with sexual TRB and increased risk for HIV among SMM. As such, interventions which target stress responses may be particularly useful for HIV risk reduction among substance using SMM (SUSMM) living with HIV (LWH). Positive affect and mindfulness-based interventions have shown promise in reducing stress across various populations living with chronic illness, including HIV. However, despite the fact SMM represent a majority of the U.S. HIV+ population and SUSMM in particular experience prominent, unique stressors, few studies have examined these interventions and their potential health benefits for SUSMM-LWH. This R34 application builds on our pilot work in the areas of positive affect induction and mindfulness as potential intervention approaches to improve HIV-related health outcomes among SMM-LWH. In our proof-of-concept pilot (n=22), we adapted an integrative positive affect and mindfulness intervention for mobile app delivery. Our pilot findings suggest the mobile app is an acceptable and feasible delivery platform for SMM-LWH. However, SUSMM-LWH experience unique stressors (e.g. substance use stigma), and experience additional barriers to intervention. While the in-person version of this intervention demonstrated efficacy in a randomized control trial with SUSMM-LWH, we have yet to assess acceptability and feasibility for delivering it via mobile app with this population. We propose to first pilot our existing app-based intervention with a sample of SUSMM-LWH to gather community feedback, which will be used to tailor the app design and intervention content for the target population. In Phase 2, we will pilot a factorial optimization trial to assess acceptability and feasibility of two additional features that may enhance our just-in-time-adaptive ecological momentary intervention (EMI) design and delivery: (1) random craving prompts throughout the day in addition to a

fixed, once-daily survey and (2) a smart watch in addition to our phone-based EMI. Data from this formative pilot will inform the development of subsequent research grant application to conduct a larger factorial optimization trail to assess how each of these new features (and their combination) impact the effectiveness of our intervention. This will be followed by a subsequent randomized control trial to evaluate efficacy of our optimized intervention, which will have a powerful and sustained effect on substance use and other HIV-related outcomes for SUSMM-LWH.

PUBLIC HEALTH RELEVANCE: In the U.S., the majority of individuals living with HIV (LWH) are sexual minority men (SMM) for whom sexual minority and HIV-related stress add to general life stressors to increase health risks, including substance use and other HIV-related risk. Positive affect and mindfulness, as stress buffering mechanisms, have shown promise for improving substance use and other HIV-related health outcomes in this population. We propose to tailor and optimize our integrative positive affect and mindfulness, app-based intervention for SMM-LWH and comorbid substance use, which will have a powerful and sustained impact on HIV-related health outcomes for this population.

CRITIQUE 1

Significance: 2
Investigator(s): 2
Innovation: 2
Approach: 2
Environment: 1

Overall Impact: This revised proposal is highly responsive to the prior review, addressing perceived deficiencies in the approach or using pilot data to successfully argue to retain proposed components. The proposal aims to adapt an app based positive affect and mindfulness intervention for sexual minority men living with HIV for use among substance using SMM-LWH and to use MOST to fine-tune the intervention and assess acceptability and feasibility of two components of the intervention: random daily assessments in addition to fixed assessments and the use of a smartwatch in addition to the phone to facilitate EMA and EMI. The MPIs are early career investigators who have been involved in this line of research and bring extensive experience in all aspect of the proposed study. They are also supported by more senior Co-Is and consultants with a solid collaboration history. The study is innovative in terms of the intervention approach as well as the methodology. The approach is rigorous and has been significantly improved by the addition of objective measures of adherence, analysis of passive data from the smartwatch. Only minimal weaknesses detract from this highly significant and innovative study which has high potential to have a high impact on increasing treatment adherence among SUSSM LWH.

1. Significance:

Strengths

- The study focuses on a population in need of interventions to improve treatment adherence and who face significant challenges.
- A low burden intervention that does not demand extensive human resources to deliver
- The study builds upon a series of successful pilot studies that support this adaptation of the intervention.

- The addition of objective markers of adherence and substance use to substantiate self-reports
- The addition of passive data collection and analysis from the smartwatch
- The addition of preliminary analyses of sexual risk behavior, substance use, and treatment adherence outcomes
- Remote delivery of evidence-based interventions is a high priority

Weaknesses

- Many substance using SMM will not be able to afford smartwatches

2. Investigator(s):

Strengths

- Given their prior work in this area, this team is uniquely qualified to conduct this study.
- Drs. Sizemore and Millar may be early career investigators but have impressive experience for this stage in their career and have the requisite experience to conduct this study
- Dr. Rendina is available to support Drs. Sizemore and Millar during the conduct of the study.
- Strong history of prior collaboration among team members
- Consultants Moskowitz and Carrico developed the earlier versions of the interventions being adapted.
- Dr. Carrico brings expertise in stimulant-using SMM

Weaknesses

- None noted

3. Innovation:

Strengths

- The JITAI EMI for substance using SMM is innovative
- Integration of Community-based research, Scrum, and MOST to form the intervention
- The integration of a micro-randomized trial in a factorial optimization trial

Weaknesses

- Although the proposed intervention includes new components, many aspects have been previously developed and tested

4. Approach:

Strengths

- Based on prior studies and an existing JITAI EMI
- The integration of the CAB to optimize the delivery of the intervention
- Study appropriately focuses on optimization of the components prior to an R01 and on assessing feasibility and acceptability
- Multiple data sources, quantitative and qualitative, subjective and objective, and passive

Weaknesses

- No details are offered about the possible areas of inquiry in the IDIs

5. Environment:

Strengths

- PRIDE has an extensive research portfolio and excellent resources to conduct the proposed study

Weaknesses

- None noted

Study Timeline:

Strengths

- Timeline is very detailed
- Feasible to the proposed study activities

Weaknesses

- None noted

Protections for Human Subjects:

Acceptable Risks and/or Adequate Protections

- Adequate protections for a minimal risk study

Data and Safety Monitoring Plan (Applicable for Clinical Trials Only):

Acceptable

Inclusion Plans:

- Sex/Gender: Distribution justified scientifically
- Race/Ethnicity: Distribution justified scientifically
- For NIH-Defined Phase III trials, Plans for valid design and analysis: Not applicable
- Inclusion/Exclusion Based on Age: Distribution justified scientifically
- Inclusion/exclusion criteria justified scientifically

Vertebrate Animals:

Not Applicable (No Vertebrate Animals)

Biohazards:

Not Applicable (No Biohazards)

Resubmission:

- This resubmission is highly responsive to the prior review, addressing the key issues raised by the prior reviewers.

Resource Sharing Plans:

Acceptable

Budget and Period of Support:

Recommend as Requested

CRITIQUE 2

Significance: 3
Investigator(s): 1
Innovation: 3
Approach: 4
Environment: 1

Overall Impact: This R34 submission proposes to tailor an existing app to deliver an intervention for sexual minority men who are living with HIV and use substances. Intervention components incorporate mindfulness and positive affect to target stress. The app will then be pilot tested through a 2x2 design to evaluate feasibility and acceptability of intervention components that will be incorporated to improve compliance/engagement, especially for substance users. Revisions build upon strengths of the original proposal that included strong preliminary work (the app has been pilot tested with sexual minority men living with HIV), a multidisciplinary team with relevant expertise, solid plans to tailor the intervention through a community-centered approach based on Scrum and evaluate intervention components through a 2x2 factorial design. Investigators were largely responsive to reviewer critiques, especially in clarifying study design (e.g., frequency of craving prompts) and measures (e.g., passive Fitbit data). There was one set of revisions that did not go far enough. In responding to a reviewer comments, the collection of adherence/substance use data was added and collection of passive smart watch data was clarified. This was good, along with the addition of outcome analyses briefly discussed in D9. No discussion was added to the aims, even for preliminary/exploratory outcome analyses. In their reviewer responses, the investigators provide another unsatisfactory statement that a focus on feasibility/acceptability is within the scope of an R34. This is true, but the data will be collected and analyzed based on what is indicated in the proposal.

1. Significance:

Strengths

- People living with HIV are impacted by stressful life events more than individuals in the general population.
- Stress is linked to HIV progression and HIV transmission behaviors.
- Sexual minority men are disproportionately impacted by HIV, especially those that are substance users.
- Interventions that address mindfulness and positive affect can reduce stress and, in turn, reduce substance use.

- Proposed work addresses a number of NIDA's funding priorities, notably the development of brief evidence-based interventions to reduce substance use among people living with HIV.

Weaknesses

- Proposed work is logical in building on established work in populations with overlapping characteristics but also incremental.
- Question if an intervention that requires app usage and wearing a Fitbit will be suitable for the most marginalized sexual minority men living with HIV.

2. Investigator(s):

Strengths

- Strong multidisciplinary research team with relevant expertise in HIV research, intervention development, substance use, mHealth and EMA research.
- MPI are young investigators supported by seasoned researchers.

Weaknesses

- None noted by reviewer.

3. Innovation:

Strengths

- MOST framework is still somewhat innovative, especially in combination with Scrum Agile framework adapted from software development.
- JITAI evaluated within factorial design.
- Behavioral intervention for substance-using sexual minority men living with HIV delivered on mobile phone.

Weaknesses

- None noted by reviewer.

4. Approach:

Strengths

- Solid foundation of preliminary work that includes prior evaluation of the positive affect/mindfulness intervention delivered in person through an RCT and pilot testing of a mobile phone-based version of the intervention with sexual minority men living with HIV.
- Community-based engagement that involves a CAB and multiple focus groups using Scrum framework.
- MOST framework that includes 2x2 factorial design to evaluate intervention components to improve engagement/compliance – random prompts and passive data collection through a Fitbit.
- App-based JITAI activities selected through micro-randomization.
- Internet recruitment through data websites and social media to streamline recruitment.

- Thorough collection of HIV, substance use and stress measures assessed through EMA, baseline/follow-up surveys, and timeline followback.
- In response to reviewer critiques, measurement of feasibility and acceptability has been clarified, as well as cut offs to determine success and rationale.

Weaknesses

- In response to a reviewer critique, blood draws will be conducted and hair samples will be collected. Collection of passive smart watch data was also clarified. D9 briefly discusses preliminary outcome analyses. Yet, none of this data, which could shed light on preliminary efficacy and be interesting, will be analyzed as part of the aims. The investigators provide an awkward justification in their response to reviewers is that the focus on feasibility/acceptability is aligned with R34 aims.
- Minimal details are provided on how preliminary outcome data in D9 will be analyzed, e.g. types of analyses applied to Fitbit biometric data to tease out time series patterns.

5. Environment:

Strengths

- Promoting Resilience, Intersectionality, Diversity, and Equity in Health Research Consortium at Hunter College supported preliminary work and will provide infrastructure for the proposed work.

Weaknesses

- None noted by reviewer.

Study Timeline:

Strengths

- Appropriate details for administrative and research activities.

Weaknesses

- None noted by reviewer.

Protections for Human Subjects:

Acceptable Risks and/or Adequate Protections

- Adequate consideration benefits/risks. Minimal risk anticipated.

Data and Safety Monitoring Plan (Applicable for Clinical Trials Only):

Not Applicable (No Clinical Trials)

Inclusion Plans:

- Sex/Gender: Distribution justified scientifically
- Race/Ethnicity: Distribution justified scientifically
- For NIH-Defined Phase III trials, Plans for valid design and analysis: Not applicable
- Inclusion/Exclusion Based on Age: Distribution justified scientifically

- No race/ethnicity exclusion criteria. Sexual minority men, 18 years and older justified by focus of the intervention.

Vertebrate Animals:

Not Applicable (No Vertebrate Animals)

Biohazards:

Not Applicable (No Biohazards)

Resubmission:

- The investigators were largely responsive to reviewer critiques, especially in clarifying study design (e.g., frequency of craving prompts and who was on the CAB) and measures (e.g., passive Fitbit data). They also restricted substance use to stimulant use to address concerns about including sexual minority men living with HIV who used any substances.

Resource Sharing Plans:

Acceptable

Authentication of Key Biological and/or Chemical Resources:

Acceptable

Budget and Period of Support:

Recommend as Requested

CRITIQUE 3

Significance: 1
Investigator(s): 1
Innovation: 1
Approach: 2
Environment: 1

Overall Impact: This R34 resubmission aims to tailor an existing stress reduction app for substance-using sexual minority men (SUSMM). The study is both significant and innovative in numerous ways. Investigators have been responsive to requests for revisions and further information in the initial submission and as a result the proposal has been significantly improved. There is only relatively minor feedback on this overall extremely well-written and well-executed revision.

1. Significance:

Strengths

- Individuals living with HIV are disproportionately impacted by stress.

- Due to the accumulation of intersecting stigma and stress, SMM-LWH experience health disparities relative to others.
- Stress is adversely linked to a range of HIV-related outcomes, as well as increased substance use cravings.
- Mobile health apps are highly accessible and can be used to successfully deliver ecological momentary interventions (EMI).
- PI's evidence-based intervention has been piloted for use with mobile app delivery.

Weaknesses

- None noted

2. Investigator(s):

Strengths

- PI Dr. Sizemore is an ESI who is embedded within a high capacity research lab setting at Hunter College. They have led the study team in the adapting intervention content for the current study.
- MPI Dr. Milar brings extensive experience and collaboration with the study team.
- Dr. Rendina is a senior scientist who will have the ability to provide oversight over all aspects of the study.
- Consultants Drs. Moskowitz and Carrico bring expertise in stress and mindfulness, as relevant to the proposed project.

Weaknesses

- None noted

3. Innovation:

Strengths

- Investigators have adapted the intervention for mobile app delivery, using a JITAI design and EMI delivery, as recommended by NIDA and others.
- The study will be informed by a community-engaged approach, together with components form other social science and software development theories and frameworks.
- Investigators are evaluating the feasibility of a micro-randomized trial, embedded within a factorial optimization trial.

Weaknesses

- None noted

4. Approach:

Strengths

- In response to the very thorough prior review, investigators have been highly responsive to recommendations and critiques. In response to requests for biometric data and smart equipment, they will now use a blood draw and hair sample at Baseline and Follow-Up, and also integrated smart watches.

- Rather than “any substance use,” investigators will limit inclusion criteria to SMM-LWH reporting stimulant use only – participants must report at least five days of stimulant use in the past 3 months.
- Investigators have clarified the 90-day length of participation in the project

Weaknesses

- The role and details of involvement of the Community Advisory Board are still relatively unclear, given all other moving pieces of this relatively complex study.

5. Environment:

Strengths

- CUNY/Hunter provide a solid and high capacity environment for accomplishing the study aims.

Weaknesses

- None noted by reviewer

Protections for Human Subjects:

Acceptable Risks and/or Adequate Protections

Data and Safety Monitoring Plan (Applicable for Clinical Trials Only):

Not Applicable (No Clinical Trials)

Inclusion Plans:

- Sex/Gender: Distribution justified scientifically
- Race/Ethnicity: Distribution justified scientifically
- For NIH-Defined Phase III trials, Plans for valid design and analysis: Not applicable
- Inclusion/Exclusion Based on Age: Distribution justified scientifically

Vertebrate Animals:

Not Applicable (No Vertebrate Animals)

Biohazards:

Not Applicable (No Biohazards)

Resubmission:

Investigators have been responsive to requests for revisions and further information in the initial submission and as a result the proposal has been significantly improved.

Resource Sharing Plans:

Not Applicable (No Relevant Resources)

Budget and Period of Support:

Recommend as Requested

THE FOLLOWING SECTIONS WERE PREPARED BY THE SCIENTIFIC REVIEW OFFICER TO SUMMARIZE THE OUTCOME OF DISCUSSIONS OF THE REVIEW COMMITTEE, OR REVIEWERS' WRITTEN CRITIQUES, ON THE FOLLOWING ISSUES:

PROTECTION OF HUMAN SUBJECTS: ACCEPTABLE

INCLUSION OF WOMEN PLAN: ACCEPTABLE

INCLUSION OF MINORITIES PLAN: ACCEPTABLE

INCLUSION ACROSS THE LIFESPAN: ACCEPTABLE

COMMITTEE BUDGET RECOMMENDATIONS: The budget was recommended as requested.

Footnotes for 1 R34 DA053999-01A1; PI Name: Sizemore, Kayla Marie

NIH has modified its policy regarding the receipt of resubmissions (amended applications).See Guide Notice NOT-OD-18-197 at https://grants.nih.gov/grants/guide/notice-files/NOT-OD-18-197.html. The impact/priority score is calculated after discussion of an application by averaging the overall scores (1-9) given by all voting reviewers on the committee and multiplying by 10. The criterion scores are submitted prior to the meeting by the individual reviewers assigned to an application, and are not discussed specifically at the review meeting or calculated into the overall impact score. Some applications also receive a percentile ranking. For details on the review process, see http://grants.nih.gov/grants/peer_review_process.htm#scoring.
